# An application of the Shapley value to the analysis of co-expression networks

**DOI:** 10.1007/s41109-018-0095-y

**Published:** 2018-08-24

**Authors:** Giulia Cesari, Encarnación Algaba, Stefano Moretti, Juan A. Nepomuceno

**Affiliations:** 1 Department of Mathematics, Politecnico di Milano, Milano, Italy; 2 Department of Applied Mathematics and IMUS, University of Seville, Seville, Spain; 3 Université Paris-Dauphine, PSL Research University, CNRS, LAMSADE, Paris, 75016 France; 4 Department of Computer Languages and Systems, University of Seville, Seville, Spain

**Keywords:** Cooperative game theory, Centrality, Co-expression networks, Shapley value

## Abstract

We study the problem of identifying relevant genes in a co-expression network using a (cooperative) game theoretic approach. The Shapley value of a cooperative game is used to asses the relevance of each gene in interaction with the others, and to stress the role of nodes in the periphery of a co-expression network for the regulation of complex biological pathways of interest. An application of the method to the analysis of gene expression data from microarrays is presented, as well as a comparison with classical centrality indices. Finally, making further assumptions about the a priori importance of genes, we combine the game theoretic model with other techniques from cluster analysis.

**Electronic supplementary material:**

The online version of this article (10.1007/s41109-018-0095-y) contains supplementary material, which is available to authorized users.

## Introduction

A *co-expression network* is an undirected graph where the nodes correspond to the genes, and a link between two genes is established if the two genes have a “similar” expression profiles in a dataset (Zhang and Horvath 2005; Parmigiani et al. 2003; Markowetz and Spang 2007). Over the last two decades, centrality analysis (Freeman 1978; Koschützki et al. 2005) was successfully used to measure the role played by each gene to influence the very complex system of genes’ relationships in a co-expression network. For instance, some independent works (Bergmann et al. 2004; Carlson et al. 2006) have shown that in co-expression networks genes with high degree-centrality are also likely to be *essential*, i.e. critical for the survival of different organisms. In a similar way, in the paper (Giorgi et al. 2013) it was shown that betweenness centrality, another measure of the nodes centrality, is in general a positive marker for essential genes in *Arabidopsis thaliana*. Other examples of application of centrality measures for the analysis of genetic networks can be found in the papers (Jeong et al. 2001; Junker et al. 2006 and Zampetaki et al. 2010).

On the other hand, in co-expression networks, genes are governed by complex regulatory mechanisms and the effects on the cell can be appreciated only if many genes simultaneously change their expression behaviour. For this reason, it seems valuable to conceive centrality notions taking into account not only the contribution of single nodes to the whole structure of the network, but also the role played by each node to all possible levels of interaction. To this aim, cooperative game theory was recently proposed as a theoretical framework for the design of centrality measures keeping into account the interactions among genes in subgroups or coalitions. For example, relevance indices based on coalitional games have been successfully applied to different kinds of biological networks, such as *brain networks* (Kaufman et al. 2005; Keinan et al. 2004; Kötter et al. 2007), *gene networks* (Moretti et al. 2010), and *metabolic networks* (Sajitz-Hermstein and Nikoloski 2012), and for the analysis of different biological data (Sajitz-Hermstein and Nikoloski 2013; Fagnocchi et al. 2015).

In this paper, we apply a game theoretic index recently introduced in the paper (Cesari et al. 2017) to identify the most relevant genes in a co-expression network. Such an index generalizes the notion of degree centrality, whose correlation with the essential genes for different biological systems is supported by several studies (see Bergmann et al. (2004), Carlson et al. (2006), Jeong et al. (2001), Junker et al. (2006),Zampetaki et al. (2010)). First, we define a specific cooperative game, where the players are the genes and the worth of a set of genes depends on the structure of the co-expression network and a parameter that specifies the a priori importance of each gene. Then, we use the Shapley value (Shapley 1953) of a cooperative game to quantify the potential of a gene in preserving the regulatory activity across all possible subsets of genes in a co-expression network. In the paper (Cesari et al. 2017) we used the axiomatic approach in order to justify the use of the Shapley value as a centrality measure, i.e., we proved that the Shapley value is the unique index that satisfy a set of properties with a precise meaning in the context of co-expression networks. In this paper, our objective is to show that the ability of the Shapley value to single out relevant genes in a co-expression network from the literature, is comparable to the one of other classical centrality measures. At the same time, we show that the information provided in terms of genes selected by the Shapley value is complementary to the information provided by the other measures. In other words, this paper is devoted to the application and the validation of the relevance index introduced in the paper (Cesari et al. 2017), and its comparison with other classical centrality indices.

In order to validate the use of the relevance index on a real gene expression dataset related to lung cancer disease (Landi et al. 2008), three relevance analyses are performed, for different choices of the genes’ weights: first, no a priori knowledge is assumed, i.e. all genes are assigned the same weight; secondly, a list of known oncogenes is taken into consideration by dividing the set of genes in key-genes and non-key-genes and lastly, the game-theoretical approach is combined with clustering analysis in order to assess the relevance of genes in the network. A comparison among the three analyses, as well as a comparison of Shapley value of specific coalitional games with classical centrality indices is presented and the results are investigated from a biological point of view.

The paper is structured as follows. “A motivating example” section presents a motivating example, in order to clarify the significance and scope of the Shapley value and the difference with respect to classical centrality measures. In “Methodology” section we introduce the methodology, describing the game-theoretical relevance index and its interpretation as a centrality measure. An application to gene expression data from microarray technology is presented in “Experimental results” and “Conclusions” sections. The lists of genes selected by the Shapley value are provided as additional files.

## A motivating example

In some cases, classical centrality measures may yield inaccurate or misleading results. As an example, in the paper (Gaitieri and Sibille 2011) it was shown that differentially expressed genes in major depression (i.e. those genes that present a statistically different behaviour in depressed patients compared to healthy patients) reside in the periphery of resilient gene co-expression networks, thus suggesting that the “central” genes are not always the most relevant in the regulatory processes within gene networks. In the paper (Mar et al. 2011), the authors have reported the tendency of genes with higher expression variance to have fewer connections across signalling networks. Moreover, in the paper (Kim et al. 2007), it was observed that proteins that have been under positive selection are located at the periphery of the interaction network.

**Fig. 1:**
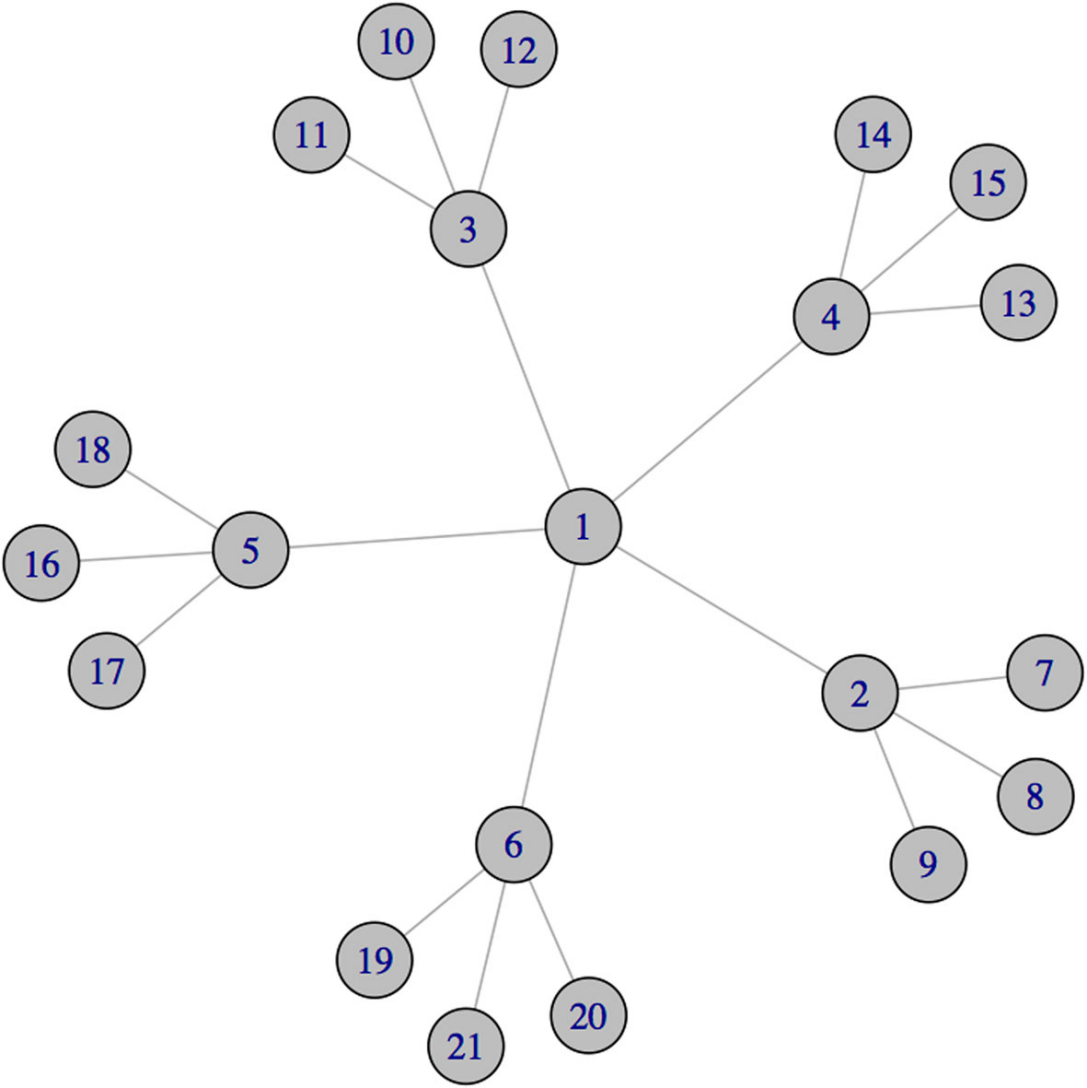
A network with 21 nodes
- the removal (or inhibition) of some of these nodes breaks down the regulatory activity of the network, by leaving all the leaf nodes isolated.

For instance, consider the graph depicted in Fig. 1. Classical centrality measures (precisely, the *degree centrality* (Nieminen 1974; Shaw 1954), the *closeness centrality* (Beauchamp 1965; Sabidussi 1966), the *betweenness centrality* (Bavelas 1948; Freeman 1977) and the *ei genvector centrality* (Bonacich 1972); see “Classical centrality measures” section for a formal definition of these measures) assign the highest relevance to node 1. In fact, node 1 has the maximum *degree* (i.e., number of neighbours), it is the node with the shortest average distance from all the other nodes in the graph (closeness centrality), it lies on the highest number of shortest paths connecting all pairs of other nodes (betweenness centrality), and it is directly connected with many central nodes (eigenvector centrality). On the other hand, the nodes 2,…,6 share two interesting characteristics that make them relevant when the network depicted in Fig. 1 represents a co-expression network:

- through their connections, these nodes are able to influence the expression of all the other genes in the network, i.e. they interact, directly or via node 1, with all the other genes within the network;

In the remaining of this paper, we introduce and discuss an application of the Shapley value aimed at measuring the potential of a gene in preserving the regulatory activity within a co-expression network. On a co-expression network built over a dataset from the literature, we show that the Shapley value of the coalitional game introduced in the paper (Cesari et al. 2017) can be interpreted in terms of the ability of genes to absorb the effects of the inhibition of other correlated genes. Stated differently, we show that the Shapley value highlights the role of genes in the overall “connectivity” of a co-expression network, by taking into account the effect that their removal has over the induced sub-networks. In this sense, in Fig. 1, node 2 (as well as nodes 3,4,5 and 6) is more relevant than node 1: when node 1 is removed, the network is divided into five components, whose overall regulation is maintained thanks to the presence of nodes 2, 3, 4, 5 and 6 respectively. On the other hand, when one of these last nodes is removed, the network is split in four component, three of which are no longer able (as being isolated nodes) to maintain their regulatory activity. With the objective to provide an index aimed at representing this type of relevance for genes in a co-expression network, in the following we consider a coalitional game where the value of a coalition of genes depends on the cardinality of the coalition itself and of its neighbourhood. The more the genes that are directly interacting in the network with genes in the coalition, and therefore the ability of the coalition to keep the network connected, the higher the strength of the coalition. Following the approach introduced in the paper (Cesari et al. 2017), we propose the Shapley value of such a coalitional game as a relevance index for genes in co-expression networks, taking into account the marginal contributions of genes to the connectivity of all the coalitions of genes in the network. We use the Shapley value to assess the relevance of genes in a real co-expression network related to lung cancer, by means of three different analyses. On such a network, when no a priori knowledge is assumed about the genes under analysis (see, for instance, the first analysis in “First analysis” section), the Shapley value is able to highlight the role of genes in the overall connectivity of the network, by assigning the highest relevance to those genes that share the two aforementioned characteristics. We argue that this interesting behaviour of the Shapley value to single out nodes that may break down the regulatory activity of the network holds in general for sparse graphs characterized by a relative low number of cycles; whereas in graphs where the peripheral nodes belong to more connected components or clusters, the indication provided by a high Shapley value seems more related to the role of certain genes to mediate the regulation between a cluster and the other structures of the network (this point will be further discussed in Example 2).

## Methodology

### Classical centrality measures

An undirected *graph* or *network* is a pair 〈*N*,*E*〉, where *N* is a finite set of *vertices* or *nodes* and *E* is a set of edges *e* of the form {*i*,*j*} with *i*,*j*∈*N*, *i*≠*j*.

We define the set of *neighbours* of a node *i* in graph 〈*N*,*E*〉 as the set *N*_*i*_(*E*)={*j*∈*N*:{*i*,*j*}∈*E*}, and the *degree* of *i* as the number *d*_*i*_(*E*)=|*N*_*i*_(*E*)| of neighbours of *i* in graph 〈*N*,*E*〉. With a slight abuse of notation, we denote by *N*_*S*_(*E*)={*j*∈*N*:∃*i*∈*S* s.t.*j*∈*N*_*i*_(*E*)} the set of neighbours of nodes in *S*∈2^*N*^, *S*≠*∅*, and in the graph 〈*N*,*E*〉. A *path* between nodes *i* and *j* in a graph 〈*N*,*E*〉 is a finite sequence of nodes (*i*_0_,*i*_1_,...,*i*_*k*_), where *i*=*i*_0_ and *j*=*i*_*k*_, *k*≥1, such that {*i*_*s*_,*i*_*s*+1_}∈*E* for each *s*∈{0,⋯,*k*−1} and such that all these edges are distinct. Two nodes *i*,*j*∈*N* are *connected* in 〈*N*,*E*〉 if *i*=*j* or if there exists a path between *i* and *j* in *E*. The *length* of a path between *i* and *j* is the number of edges in the path and a *shortest path* between *i* and *j* is a path between *i* and *j* with minimum length. Let *i*∈*N* and *S*⊆*N*∖{*i*}.

Centrality measures assign to each node in a network a value that corresponds to some extent to the relevance of that node within the network structure. The four classical centrality measures considered in this paper are the following:

1. *Degree centrality* (Nieminen 1974; Shaw 1954): the degree centrality of *i*∈*N* is defined as |*N*_*i*_(*E*)|, i.e. the number of neighbours of *i* in graph 〈*N*,*E*〉. It is an index of the potential communication activity of a node.
2. *Closeness centrality* (Beauchamp 1965; Sabidussi 1966): the closeness centrality of node *i* is defined as $\frac {|N|-1}{\sum _{j \in N} {h}(i,j)}$, where *h*(*i*,*j*) is the distance between *i* e *j*, i.e. the length of the shortest path between *i* and *j*. It measures to what extent a node can avoid the control potential of the others nodes.
3. *Betweeness centrality* (Bavelas 1948; Freeman 1977): the betweenness centrality of a node *k* is defined as $\sum _{i,j \in N} b_{ij}(k)$, where $b_{ij}(k)=\frac {g_{ij}(k)}{g_{ij}}$ and *g*_*ij*_ is the number of shortest paths between nodes *i* and *j*, while *g*_*ij*_(*k*) is the number of shortest paths between nodes *i* and *j* that contain *k*. It is an index of the potential of a node for control of communication.
4. *Eigenvector centrality* (Bonacich 1972): the eigenvector centrality of a node *i* is defined as the *i*−*th* element of the principal eigenvector of the adjacency matrix *A*=(*a*_*ij*_) corresponding to 〈*N*,*E*〉, where *a*_*ij*_=1 if {*i*,*j*}∈*E* and *a*_*ij*_=0 otherwise. It assigns high centrality to nodes that are highly connected to nodes who themselves have high centrality.

### A game-theoretic relevance index

Let 〈*N*,*E*〉 be a *co-expression network*, that is a network where the set of nodes *N* represents a set of genes and the set of edges *E* describes the interaction among genes, i.e. there exists an edge between two genes if they are directly interacting in the biological condition under analysis. Moreover, let $k \in \mathbb {R}^{N}$ be a parameter vector that specifies the a priori importance or *weight* of each gene. According to Cesari et al. (2017), we define the *coalitional game*$\left (N,v_{E}^{k}\right)$, where *N* is the set of genes under study and the *characteristic function*$v_{E}^{k}$ assigns a worth to each coalition of genes *S*⊆*N* representing the overall magnitude of the interaction between the genes in *S*, which takes into account the weight (i.e., the a priori importance) of each gene directly connected to *S* in the biological network. More precisely, the map $v_{E}^{k}:2^{N} \rightarrow \mathbb {R}$ assigns to each coalition *S*∈2^*N*^∖{*∅*} the value

1 $$ v_{E}^{k}(S)=\sum_{j \in S \cup N_{S}(E)} k_{j}  $$

that is the sum of the weights associated to the genes in *S* and to the ones that are directly connected in 〈*N*,*E*〉 to some genes in *S* (by convention, $v_{E}^{k}(\emptyset)=0$) (Cesari et al. 2017). The class of games (*N*,*v*) defined according to relation (1), on some gene network *G*≡〈*V*,*E*〉 and with parameter $k \in \mathbb {R}^{N}$, is denoted by $\mathcal {EK}^{N}$.

A well-known *solution* for coalitional games is the Shapley value (Shapley 1953), which was introduced in 1953 and since then applied to a wide range of fields, including biology (Moretti and Patrone 2008). The Shapley value *ρ*(*v*) of a game (*N*,*v*) is defined as the average of marginal vectors over all |*N*|! possible orders in *Σ*_*N*_ (|*N*| is the cardinality of the set *N*). In formula

2 $$ {\rho}_{i}(v)=\sum_{\sigma \in \Sigma_{N}} \frac{m_{i}^{\sigma}(v)}{|N|!} \makebox{ for all} i \in N,  $$

where *Σ*_*N*_ is the set of all possible permutations of the elements in *N* and *σ*(*i*)=*j* means that with respect to *σ* player *i* is in the *j*-th position, and where the *marginal vector*$m^{\sigma }(v) \in \mathbb {R}^{N}$ is defined by $m_{i}^{\sigma }(v)=v(\{j \in N:\sigma (j) \leq \sigma (i)\})-v(\{j \in N:\sigma (j) < \sigma (i)\})$ for each *i*∈*N* (i.e., $m_{i}^{\sigma }(v)$ is the *marginal contribution* of player *i* to the coalition of players with lower positions in *σ*).

In the paper (Cesari et al. 2017), the authors have shown that the Shapley value is the unique *relevance index* for genes (defined as a map $\rho :\mathcal {EK}^{N} \rightarrow \mathbb {R}^{N}$) which satisfies four desired properties, namely, symmetry, the dummy player property, efficiency and star-additivity. Three out of these four properties are natural axioms borrowed from the related literature on cooperative games: the property of symmetry requires that if two genes *i* and *j* have the same a priori weight (*k*_*i*_=*k*_*j*_) and in addition, they are connected to the same set of neighbours in a network, then they should have the same relevance; the dummy player property basically implies that the relevance of a disconnected node in a network coincides with its a priori importance; the efficiency axiom determines the scale of measure, setting the sum of the relevance of all genes equal to the sum of their a priori weights. The fourth property introduced in the paper (Cesari et al. 2017) to axiomatically characterize the Shapley value on the class $\mathcal {EK}^{N}$, i.e., the star-additivity axiom, says that increasing the a priori weight of a node *i* from 0 to a positive value should affect the relevance of gene *i* and its neighbours at the same extent, for whatever graph. Consequently, reallocating the a priori importance of a node among its neighbours, the star additivity property catches the idea of measuring the ability of nodes to absorb the changes in expression of correlated genes, as previously discussed in the motivating example illustrated in “A motivating example” section.

A practical limitation inherent to most of the applications of the Shapley value is the computational burden related to its calculus. Surprisingly, in the paper (Cesari et al. 2017) the authors have shown that the Shapley value of a coalitional game $\left (N,v_{E}^{k}\right)$ can be computed according to the following much simpler relation:

3 $$ {\rho}_{i}\left(v_{E}^{k}\right)=\sum_{j \in (N_{i}(E) \cup \{i\})} \frac{k_{j}}{d_{j}(E)+1},  $$

for each *i*∈*N*.

According to relation (3), a gene connected to many genes who themselves have a low degree gets a high Shapley value (in other words, the relevance of a gene increases with the number of its neighbours having a low degree). Relation (3) also suggests that genes with a high Shapley value would be able to interact directly with the maximum number of other nodes in the network and its removal would split the network in a maximum number of connected components with few genes, or eventually constituted by isolated genes. As shown in the paper (Aadithya 2010), relation (3) can be calculated via an $\mathcal {O}(|N| + |E|)$ procedure, which makes possible its computation on very large networks (recall that on a network 〈*N*,*E*〉, calculating betweenness centrality takes $\mathcal {O}(|N| |E|)$ time using Brandes’ algorithm (Brandes 2001). We conclude this section showing the results of relation (3) applied to the motivating example in “A motivating example” section.

#### **Example 1**

Consider the gene network in Fig. 1. Suppose all the genes have the same a priori weight *k*_*i*_=1∀*i*∈*N*. Then, by relation (3) ${\rho }\left (v_{E}^{k}\right)=\left (\frac {35}{30}, \frac {56}{30},\right. \frac {56}{30},\frac {56}{30}, \frac {56}{30},\frac {56}{30}, \frac {21}{30}, \frac {21}{30}, \frac {21}{30},\frac {21}{30}, \frac {21}{30},\frac {21}{30}, \frac {21}{30},\frac {21}{30}, \frac {21}{30},\frac {21}{30}, \frac {21}{30},\frac {21}{30}, \left.\frac {21}{30},\frac {21}{30},\frac {21}{30}\right)$. Therefore, the Shapley value gives the highest relevance to nodes 2,3,4,5 and 6, followed by node 1 and the least relevance to the leaf nodes {7,…,21}. Instead, all the other classical centrality measures defined in “Classical centrality measures” section provide the following ranking: node 1 is ranked first, followed by nodes {2,3,4,5,6} in the second position and, finally, by the leaf nodes with the lowest rank.

#### **Example 2**

**Fig. 2:**
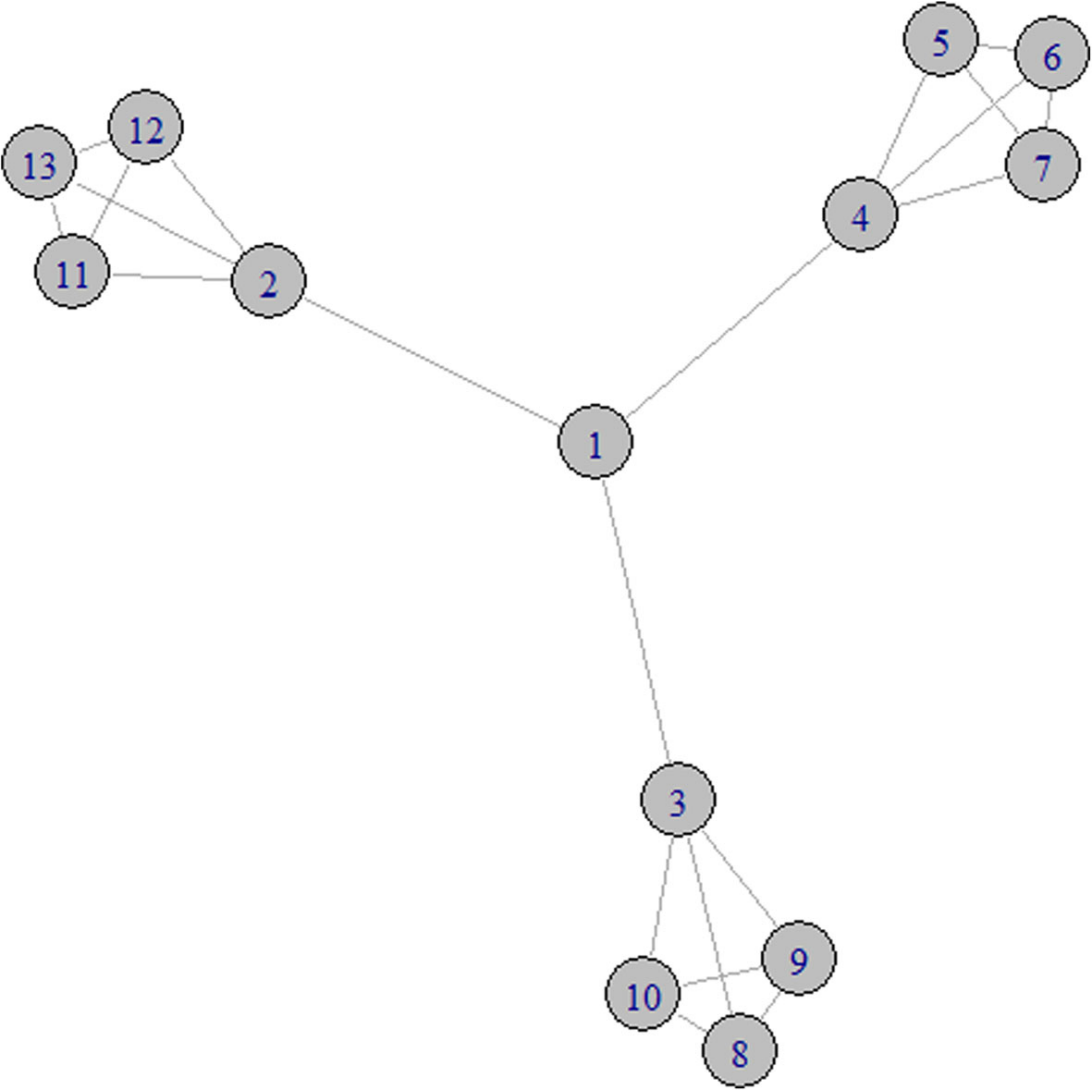
A network with 13 nodes and three cliques with four nodes each

Now, consider the gene network in Fig. 2. Suppose again that all the genes have the same a priori weight *k*_*i*_=1∀*i*∈*N*. As in Example 1, the middle position of node 1 in the graph can lead to the conclusion that 1 is the most central gene, at least, if we adopt a notion of importance related to the idea that genes influence each others via shortest paths. In fact, in the network of Fig. 2, both the closeness centrality and the betweenness centrality rank node 1 in the highest position. On the other hand, if we are interested in measuring the importance of nodes in relation to their ability to influence their neighbours, without any a priori assumption on who is influenced by whom, it seems reasonable to consider nodes 2, 3 and 4 as the most central ones, because of their role to connect each clique of four node (i.e., each subset of four nodes such that every two distinct vertices in the subject are adjacent) with the other parts of the network. So, it is not surprising that the degree centrality, the eigenvector centrality and the Shapley value give the highest relevance to such nodes. This example emphasizes the fact that divergent but still reasonable conclusions can be obtained as a logical consequence of alternative notions of relevance for nodes of a network. However, we recognize that the interpretation of the Shapley value given in the motivating example of “A motivating example” section does not apply to the graph of Fig. 2, which is characterized by multiple cliques: the removal of one of the nodes 2, 3 and 4 does not isolate any leaf node, but only fully connected components. Note also that the Shapley value is the unique measure, among the ones considered in Table 1, which ranks nodes in the cliques strictly higher than node 1 (only the degree centrality assigns the same value equal to 3 to the nodes in the cliques and to node 1). In fact, according to the Shapley value, the nodes in a clique gather some further relevance from being connected to each other, and then from being less exposed to the influence of other nodes. On the contrary, node 1 is directly connected to the very well connected nodes 2, 3 and 4, and it is exposed to their influence. On the opposite side, the betweenness centrality ranks node 1 in the top, and the nodes in the cliques in the bottom: we argue that this negative correlation between the Shapley value and the betweenness centrality is also due to the relative high number of cliques present in the network of Fig. 2, which is not the case of the other networks considered in this paper.

**Table 1:** Comparison of centrality measures in the network of Example 2

	1	2	3	4	5	6	7	8	9	10	11	12	13
*ρ*	0.85	1.20	1.20	1.20	0.95	0.95	0.95	0.95	0.95	0.95	0.95	0.95	0.95
Degree	3	4	4	4	3	3	3	3	3	3	3	3	3
Closeness	0.048	0.038	0.038	0.038	0.029	0.029	0.029	0.029	0.029	0.029	0.029	0.029	0.029
Betweenness	48	27	27	27	0	0	0	0	0	0	0	0	0
Eigenvector	0.92	1	1	1	0.79	0.79	0.79	0.79	0.79	0.79	0.79	0.79	0.79

### Related literature

Another way to keep into account the a priori importance of genes was proposed in the paper (Moretti et al. 2010) by means of the so-called *association game*, where a set of key-genes *K*⊂*N* (e.g. a set of genes known a priori to be involved in biological pathways related to chromosome damage) is considered and the value assigned to a coalition *S* is the number of key-genes interacting only with *S* formally, in the paper (Moretti et al. 2010) the value assigned to a coalition *S*⊆*N* is the cardinality of the set {*i*∈*K*:*N*_*i*_(*E*)⊆*S*}. However, the definition proposed in relation (1) seems more flexible to explore all possibilities of reciprocal influence among genes. It generalizes the game introduced in the papers (Suri and Narahari 2008; Aadithya et al. 2010) for determining the “top-*k* nodes” in a co-authorship network, by the introduction of a parameter that specifies the a priori importance of each node. The parameter vector *k* allows for an a priori ranking of the genes according to their importance, while in the previous model introduced in the paper (Moretti et al. 2010) only a two-level distinction was made between key-genes and non key-genes. Moreover, by measuring to what extent a coalition of genes is connected to the rest of the network, relation (1) generalizes the notion of degree centrality for groups of genes, which is justified by some practical evidences showing a strong correlation between the degree centrality and genes that are essential for different biological functions (see, for instance, (Bergmann et al. 2004; Carlson et al. 2006; Jeong et al. 2001; Junker et al. 2006; Zampetaki et al. 2010). In fact, if only the weight of genes inside a coalition was to be considered (and not the one of the neighbours, as in our definition), the centrality measure obtained through relation (3) would coincide with a “weighted” degree centrality.

## Experimental results

This section is devoted to the analysis of the Shapley value on co-expression networks generated from a gene expression dataset. We first introduce and discuss a preliminary analysis of the robustness of the methodology based on the Shapley value in selecting the most relevant genes.

In the following, the criterion used to establish whether two genes are co-expressed is based on the correlation between their expression profiles in the corresponding gene expression dataset (precisely, on the Pearson’s correlation coefficient). Basically, two genes are said co-expressed if and only if their Pearson’s correlation coefficient is larger than a predefined cut-off (Carter et al. 2004; Zhang and Horvath 2005). Of course, the choice of the threshold is critical for the analysis. Therefore, we start with the evaluation of the robustness of the model using alternative thresholds.

### Robustness evaluation

We tested the model on a randomly generated symmetric matrix of size 1000 with entries in the range [0,1]. To be more specific, we used a matrix where the element in row *i* and column *j* represents the correlation between gene *i* and gene *j* in a fictitious, randomly drawn dataset of 1000 genes. For the sake of this analysis the parameter vector *k* was fixed in such a way that *k*_*i*_=1 for every *i*. The matrix was transformed in a boolean adjacency matrix (where 1 represents a connection in the network and 0 means no connection) according to three different thresholds, 0.7, 0.8 and 0.9, respectively. A network was generated for each threshold according to the aforementioned criterion and the relevance index for each gene, i.e. the Shapley value *ρ* of the game defined in (1), was computed via relation (3). A comparison between the results for the three different thresholds was conducted. In particular, we selected the list of the 5% of genes with the highest Shapley value for each threshold, and we obtained the following results: 18 genes are commonly selected by the Shapley value for cutoff 0.7 and 0.8; 15 genes are commonly selected for cutoff 0.8 and 0.9 and 5 genes are commonly selected for cutoff 0.7 and 0.9 These results are summarized in Table 2.

**Table 2:** Number of common genes by using a cutoff of 0.7, 0.8 and 0.9, respectively

	0.7	0.8	0.9
0.7	50	18	5
0.8	18	50	15
0.9	5	15	50

### Relevance analysis

In this section, we present the results obtained from the application of methodology based on the Shapley value to select the most relevant genes in a lung cancer dataset according to three alternative approaches. First, the dataset is analysed by assuming no a priori knowledge of the importance of the genes in the network; secondly, the knowledge about some key oncogenes is included in the analysis and, lastly, a method from clustering analysis is used to assess the a priori importance of genes.

### Description of the dataset

We consider a gene expression dataset related with a very common kind of lung cancer called *adenocarcinoma*. Adenocarcinoma cancers are usually found in lung outer areas as the lining of the airways. This dataset with accession number GDS3257 was downloaded from the *Gene Expression Omnibus* (GEO) data base of the *National Center for Biotechnology Information* (NCBI). These data were generated in a study where 107 samples of several tumor stages in a population of smoker and non-smoker people were analyzed (Landi et al. 2008). The raw gene expression data have been preprocessed with the Babelomics tool (Medina et al. 2010) using several standard filtering steps. Concretely, those genes with a percentage of missing values greater than 80% have been removed. In the remaining of the cases, missing values have been replaced with the average of the expression profile of the row. Those gene profiles with a standard deviation smaller than 0.5 have been removed. The resulting gene expression matrix is composed by 2517 gene expression profiles (rows) and 107 samples (columns).

**Fig. 3:**
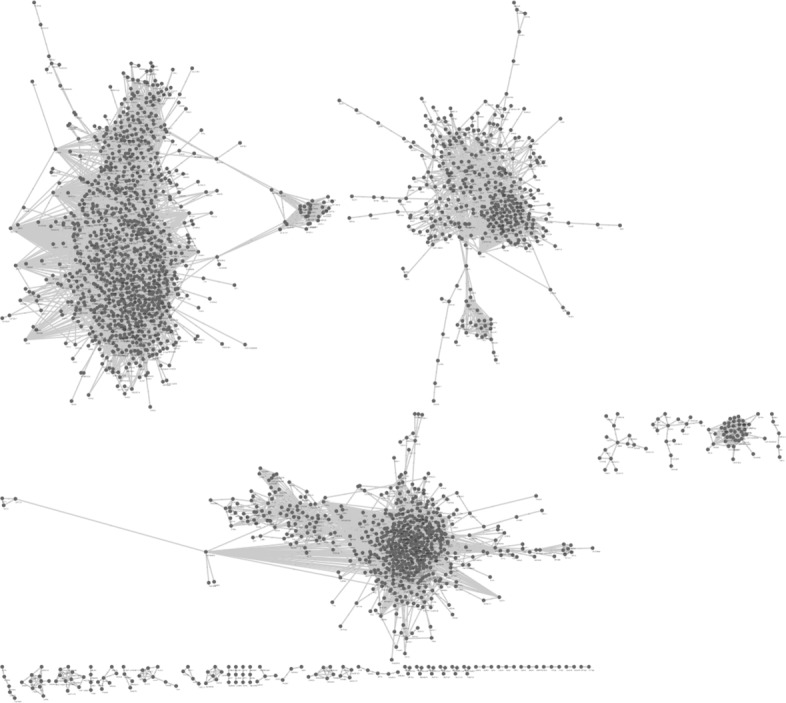
Gene co-expression network generated with lung cancer dataset

A gene co-expression network was generated, by establishing a link between two genes if and only if the Pearson’s correlation between their gene expression profiles is higher than a fixed threshold. The choice of the threshold is based on the following considerations: a suitable network should consist of connected components with the highest possible cardinality and should also be as sparse as possible in order to better reveal the relationships between the nodes (genes). Therefore, the network must be experimentally built according to an equilibrium between connectivity and sparsification (Christiano Silva and Zhao 2016). The BioLayout tool (Theocharidis et al. 2009) was used to conduct an experimental study, which led us to the choice of 0.8 as the value for the correlation threshold. The resulting network is composed by 2154 nodes (genes) and 24821 edges. Figure 3 shows a picture of the resulting network.

#### First analysis

**Fig. 4:**
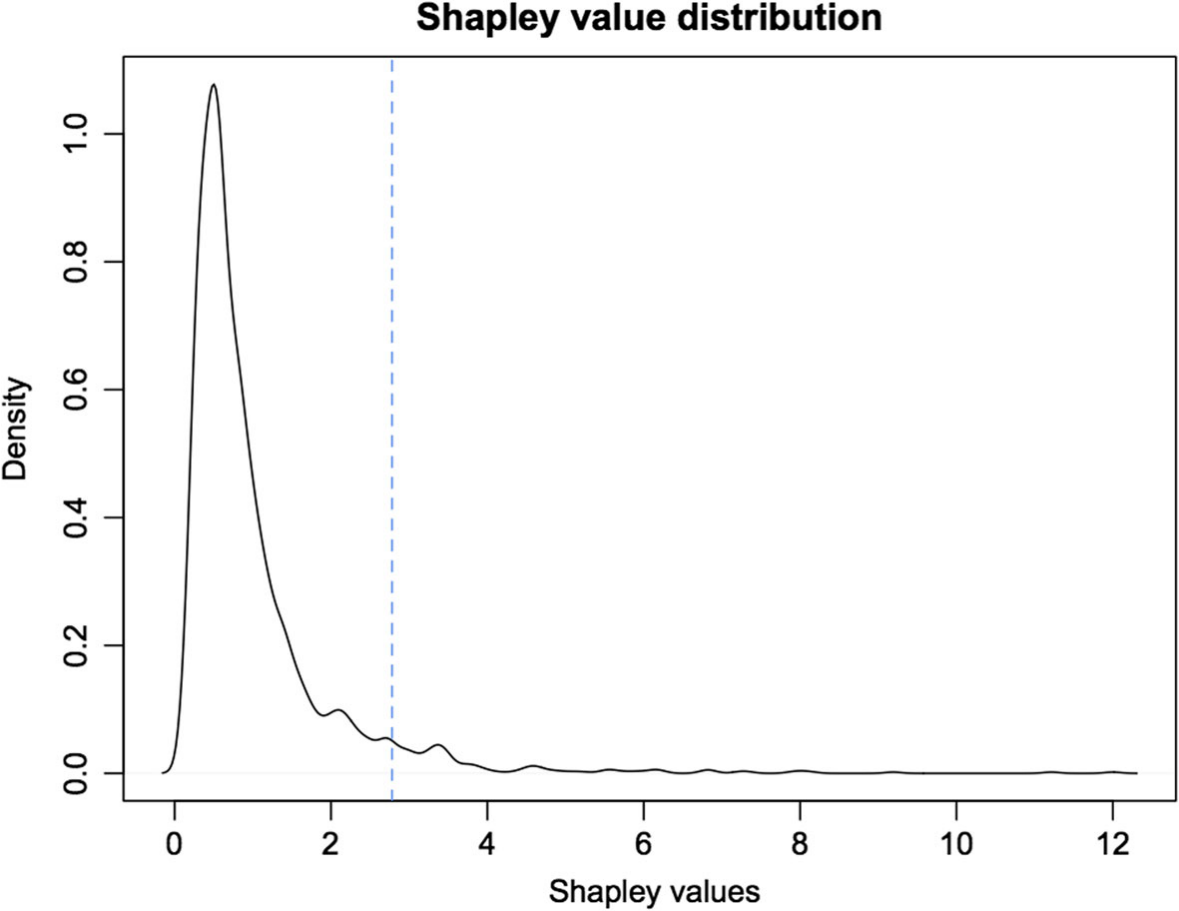
First analysis: Shapley value distribution. The density distribution of the index *ρ* is shown, for *k*_*i*_=1 for every gene *i*. The dotted vertical line represents the cutoff: the 5% of genes with highest index is selected

We carried out a first analysis on the aforementioned network, with no a priori knowledge of the importance of the different genes, i.e. setting *k*_*i*_=1 for each gene *i*∈*N*. Following this approach, the Shapley value *ρ* is computed. The density distribution of *ρ* is shown in Fig. 4.

**Fig. 5:**
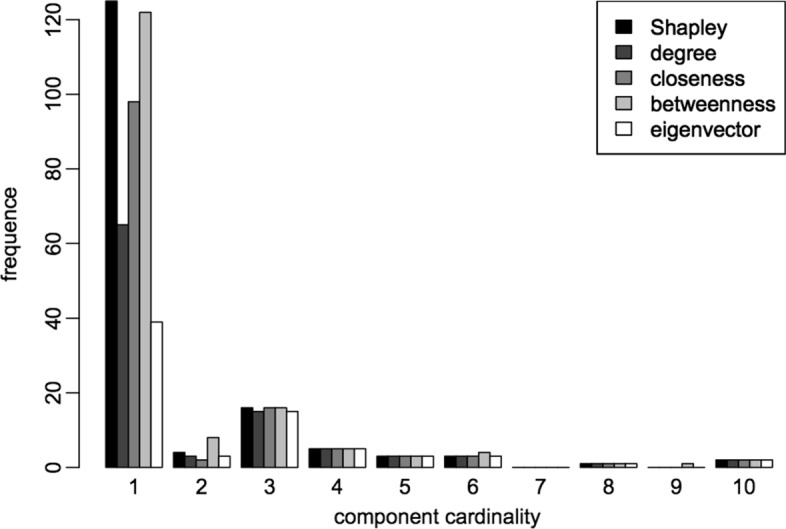
Comparison of the frequencies of components cardinality. The histogram represents the frequency of components cardinality after the removal of the genes selected by the different centrality measures

We select the 5% of genes with the highest relevance for further analysis. This list of genes is investigated with respect to the features described in the motivating example of “A motivating example” section. It turns out that, in the comparison with the classical centrality measures, the Shapley value is able to highlight these characteristics. We compared the lists of the 5% of genes with the highest value according to the different centrality measures and we obtained the following results:

(i) the 108 genes selected by the Shapley value *ρ* are directly connected to 1412 genes. With the only exception of betweenness centrality (the 108 genes selected with the highest betweenness centrality are directly connected to 1423 genes), the other measures are much less effective in this sense: the genes selected by the degree centrality interact with 1062 genes, the ones by closeness centrality with 668 and the ones by eigenvector centrality with 383 genes.
(ii) when the 108 genes selected by *ρ* are removed, the network is split in 165 connected components, 125 of which are isolated nodes. Three of these components contain a high number of genes (550, 826 and 338), another one contains 42 nodes, and the remaining ones contain very few nodes (from 2 to 10 nodes each). A similar behaviour is observed after the removal of the 108 nodes selected by the betweenness centrality: the network is split in 170 components, 122 of which are isolated nodes. On the other hand, the effects of the removal of the genes selected by the other measures are definitively less severe. See Fig. 5 for a comparison with the different measures. Note that the histogram was constructed by considering only the components with less than ten nodes, since the bigger components have very similar frequencies for all measures.

#### Second analysis

A second analysis was conducted by taking into account the presence in the network of some known lung cancer key-genes, i.e. setting *k*_*i*_=1 for each key-gene *i* and *k*_*i*_=0 otherwise. In particular, we consider a set of 23 known lung oncogenes found by mean of the *Network Cancer of Genes* tool (NCG5.0).

**Fig. 6:**
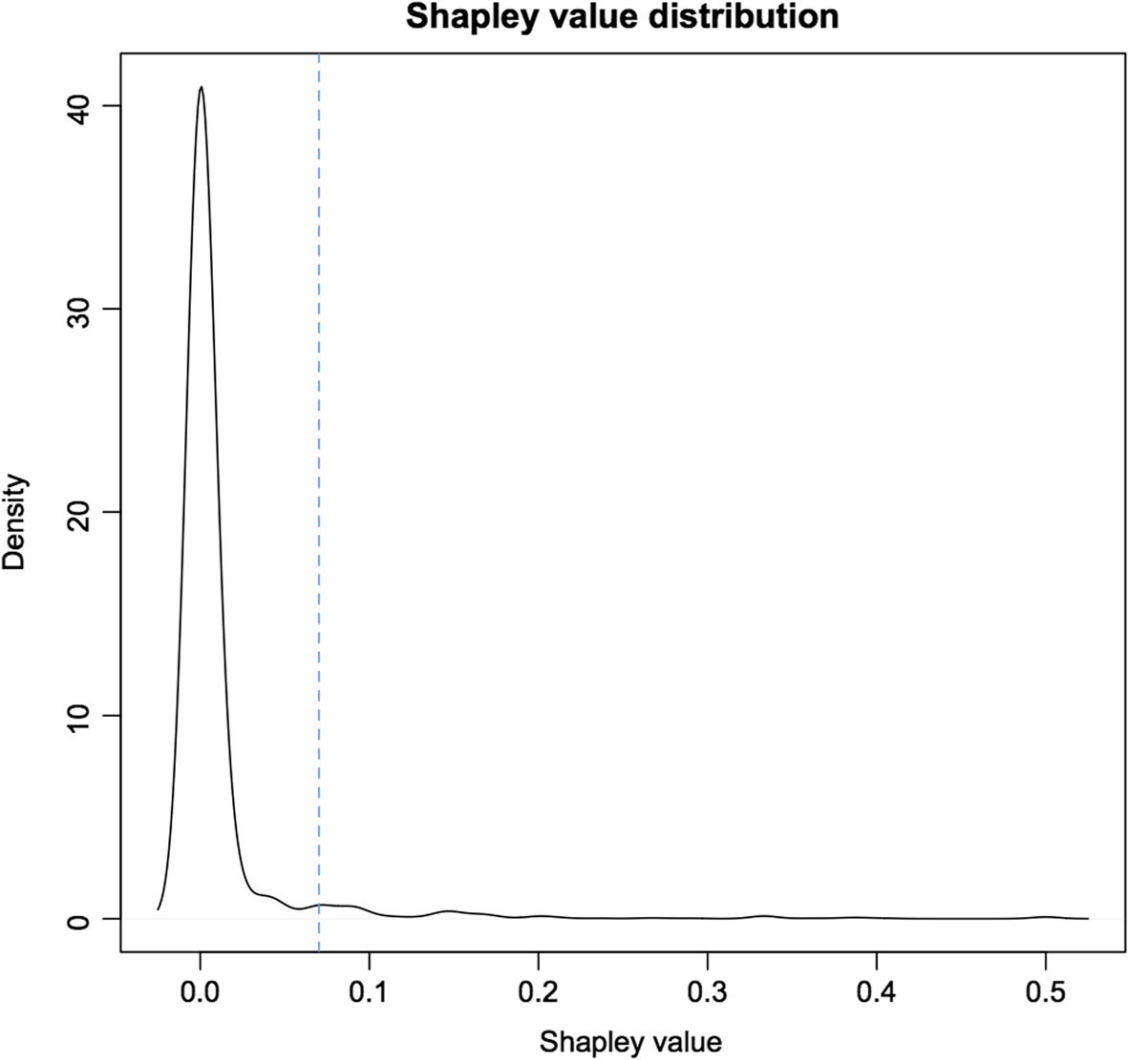
Second analysis: Shapley value distribution. The density distribution of the index *ρ* is shown, for *k*_*i*_=1 for every key-gene *i* and *k*_*i*_=0 otherwise. The dotted vertical line represents the cutoff: the 5% of genes with highest index is selected

The 5% of genes with the highest Shapley value is selected for further analysis. The density distribution of *ρ* is shown in Fig. 6.

#### Third analysis

A strength of our model relies on the possibility to integrate different tools from network analysis to assess the relevance of genes in a network. Indeed, even if the a priori weight of genes is not known, the freedom in the choice of the parameter vector *k* allows for a variety of approaches. In particular, we use some techniques from cluster analysis to define the a priori importance of genes. To be more specific, a third analysis was conducted by measuring the a priori importance of genes by a parameter vector that depends on the structure of clusters in the network. The underlying idea is the following: the a priori weight of a gene is assessed by dividing the network in clusters, through the algorithm ClusterONE (Nepusz and Yu 2012), and counting the number of clusters a gene belongs to, precisely, *k*_*i*_ for every gene *i*∈*N* is defined as the number of cluster in the network it belongs to. This approach is based on the idea (Li et al. 2008) that genes belonging to multiple clusters are to some extent important in the network.

Traditional clustering algorithms report a partition of data such that all clusters are disjoint. However, the overlapping among clusters is interesting in the context of gene interaction networks, since genes are usually involved in several processes and might, as a consequence, belong to different clusters (Li et al. 2008). ClusterONE (Nepusz and Yu 2012) is a greedy search process that finds groups of genes with a high cohesiveness among them and captures overlapping clusters of genes in a network.

**Fig. 7:**
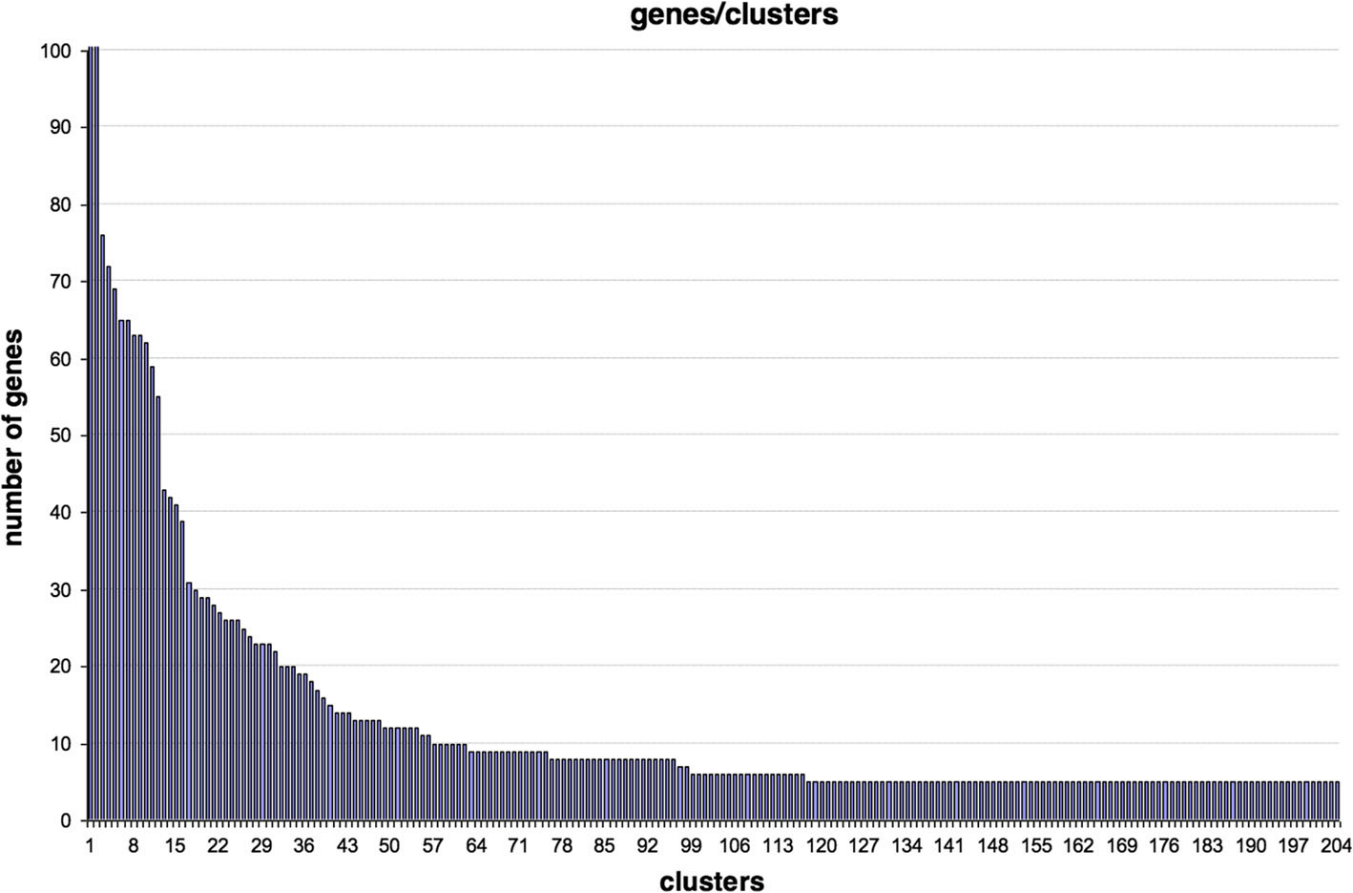
Number of genes per cluster

Precisely, we set the basic parameters for minimum cluster size and minimum cluster density, respectively, to 5 and 0.5, whereas we maintain the default values for the advanced parameters. Figure 7 shows the number of genes for each of the 204 clusters provided by the ClusterONE algorithm. Note that the first and second cluster have 306 and 107 genes, respectively. The maximum value in the *y* axis was chosen equal to 100 in order to improve the figure visualization.

**Fig. 8:**
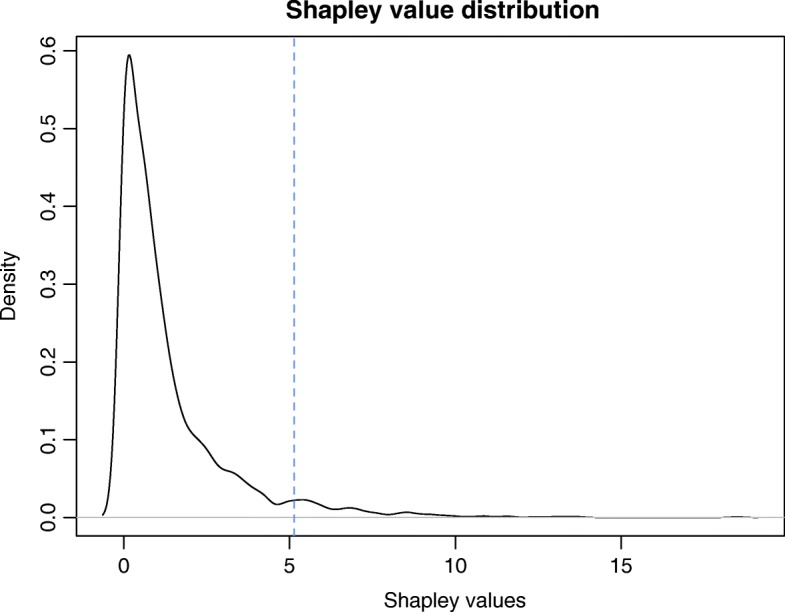
Third analysis: Shapley value distribution. The density distribution of the index *ρ* is shown, for *k*_*i*_ defined as the number of clusters gene *i* belongs to. The dotted vertical line represents the cutoff: the 5% of genes with highest index is selected

All the clusters generated through the aforementioned procedure are considered and to each gene *i* belonging to these clusters (1444 out of 2154 in the whole dataset) is assigned a weight *k*_*i*_ equal to the number of clusters it belongs to. The Shapley value is then computed and its density distribution is shown in Fig. 8. Moreover, the list of 5% of genes with the highest Shapley value is selected for further analysis.

### Results comparison

The results from the three different analyses have been compared with the results provided by the application of classical centrality measures. The number of common genes among the lists selected by different centrality measures is shown in Table 3 (the full lists of genes selected by the Shapley value for the three analyses are also provided as Additional files), and the correlation between centrality measures in terms of their Pearson correlation coefficient is provided in Table 4.

**Table 3:** Number of common genes among the relevance vectors of 108 genes provided by the different relevance measures

	*ρ*(1)	*ρ*(2)	*ρ*(3)	Degree	Closeness	Betweenness	Eigenvector
*ρ*(1)	108 (1)	27 (0.492)	61 (0.672)	49 (-0.221)	40 (0.430)	66 (0.846)	28 (0.509)
*ρ*(2)	27 (0.492)	108 (1)	33 (0.325)	22 (0.004)	20 (0.395)	19 (0.578)	12 (0.202)
*ρ*(3)	61 (0.672)	33 (0.325)	108 (1)	31 (-0.228)	49 (0.475)	55 (0.529)	10 (0.214)
Degree	49 (-0.221)	22 (0.004)	31 (-0.228)	108 (1)	19 (0.482)	28 (-0.068)	86 (0.977)
Closeness	40 (0.430)	20 (0.395)	49 (0.475)	19 (0.482)	108 (1)	48 (0.578)	0 (NA)
Betweenness	66 (0.846)	19 (0.578)	55 (0.529)	28 (-0.068)	48 (0.697)	108 (1)	7 (0.121)
Eigenvector	28 (0.509)	12 (0.202)	10 (0.214)	86 (0.977)	0 (NA)	7 (0.121)	108 (1)

The number in parenthesis represents the correlation among the lists of common genes

**Table 4:** Correlation among the lists obtained by different centrality measures

	*ρ*(1)	*ρ*(2)	*ρ*(3)	Degree	Closeness	Betweenness	Eigenvector
*ρ*(1)	1	0.265	0.808	0.694	0	0.804	0.269
*ρ*(2)	0.265	1	0.305	0.220	-0.016	0.178	0.073
*ρ*(3)	0.808	0.305	1	0.665	0.250	0.660	0.211
Degree	0.694	0.220	0.665	1	-0.005	0.456	0.790
Closeness	0	-0.016	0.250	-0.005	1	0.148	-0.145
Betweenness	0.804	0.178	0.660	0.456	0.148	1	0.067
Eigenvector	0.269	0.073	0.211	0.790	-0.145	0.067	1

Note that the correlation coefficients are computed on the entire lists of 2154 genes

The Shapley value computed according to the first analysis shows an overlap with betweenness centrality higher than the overlap of betweenness centrality with the other classical centrality measures, showing 66 genes in common (out of the 108 selected with the highest value) and a high positive correlation on the whole list of genes. In the second analysis, on the other hand, most of the genes selected by the Shapley value are not selected by other measures, with a maximum overlap of 22 genes with degree centrality, suggesting that the introduction of a priori known key-genes strongly influences the analysis towards the selection of genes that interact with this particular set of genes. On the other hand, the third analysis seems to produce results that are more similar to the ones of the first analysis, with a maximum overlap with the list selected by betweenness centrality, followed by closeness and degree centrality.

**Fig. 9:**
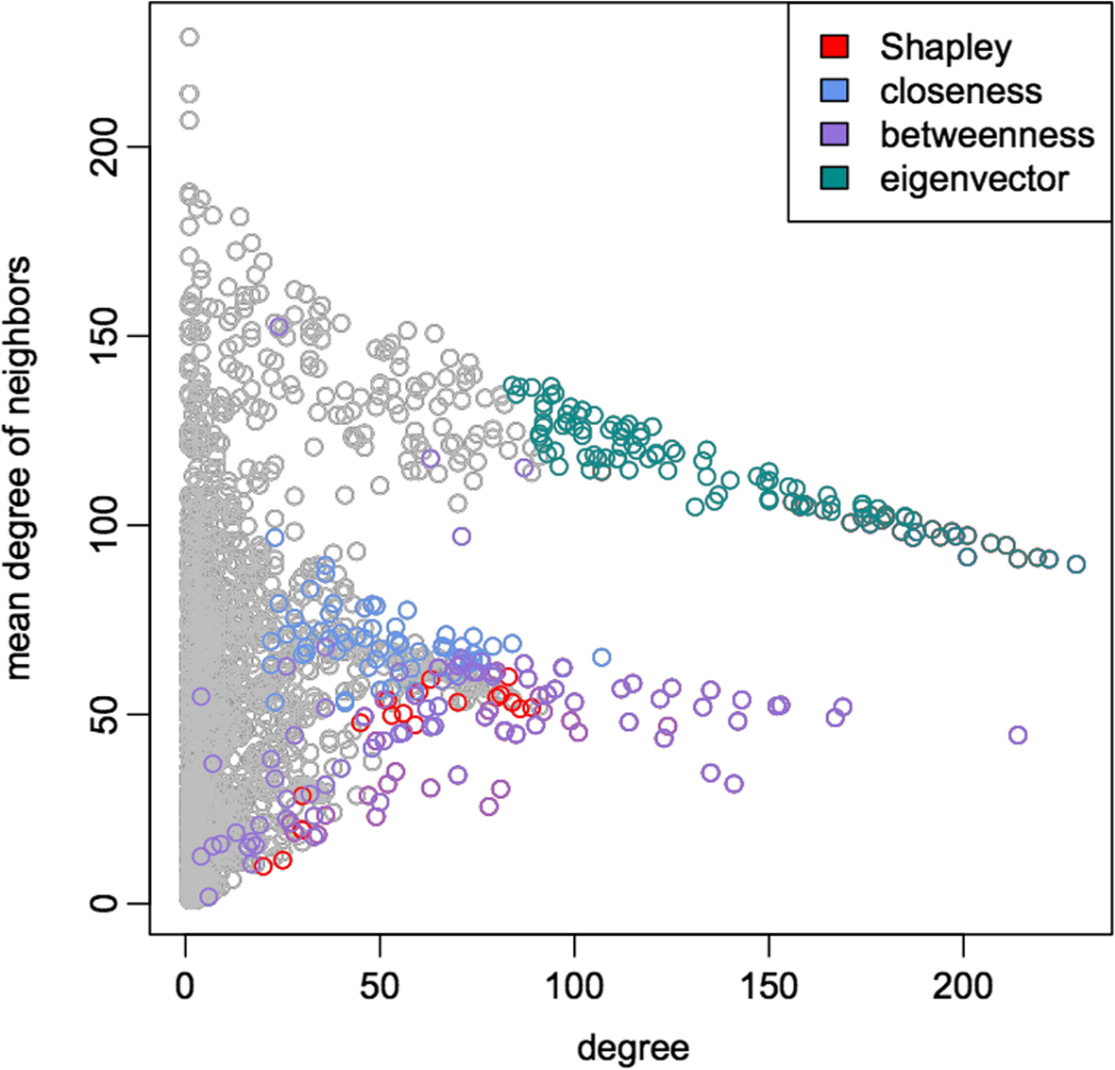
Comparison of the relation among the degree of a node and the degree of its neighbours. The points represent genes and their coordinates are given, respectively, by the degree of a gene (on the *x*-axis) and the mean degree of its neighbours (on the *y*-axis). The coloured points represent the genes selected by the different centrality measures

Moreover, all the three analyses select very few genes in common with the eigenvector centrality, which is not surprising since the Shapley value selects those genes that are co-expressed with many genes that have low degree, whereas eigenvector centrality selects genes that are highly connected to genes with high degree.The relationship among the degree of a node and the degree of its neighbours is highlighted in Fig. 9. The coloured points represent the genes selected by the different centrality measures. In particular, the red points represent the ones selected only by the Shapley value. Notice that the degree centrality selects all the nodes with degree higher than 100.

### Biological interpretation of the results

The results of the three analyses have also been investigated from a biological point of view. The number of relevant genes stored in biological repositories was considered as a quantitative criterium to compare them. First, a Literature Mining approach was used with a *Cytoscape* plug-in called *Agilent Literature Search* (Saito et al. 2012). Second, we also performed a Reactome study with the same goal. It is important to note that only the first 100 genes for each analysis in the Additional file 1: Table S1, Additional file 2: Table S2 and Additional file 3: Table S3 have been studied due to the limitations of these tools.

The Cytoscape plug-in searches a set of genes in published papers available in public repositories such as *PubMed*. The search has been performed by taking as input the list of genes selected by the Shapley value and a set of key-words, namely “Homo sapiens” and “Adenocarcinoma”. The tool provides as a result the subset of genes that are cited in the related literature.

**Fig. 10:**
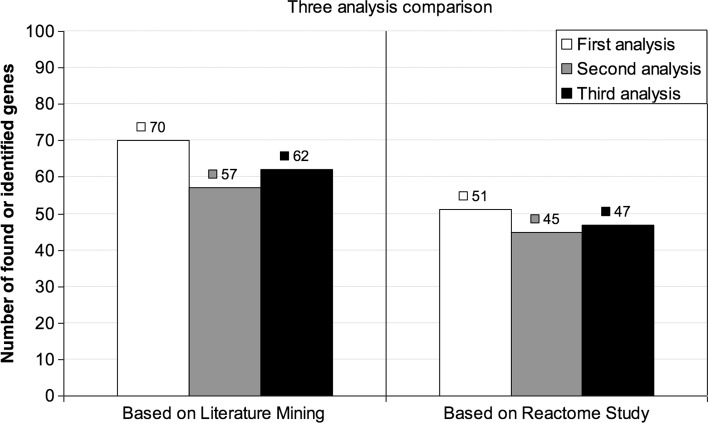
Comparison of three analysis based on Literature Mining and Reactome study

Figure 10 shows on the left-hand side the results of the Literature Mining-based comparison. The first, second and third analysis report 70, 57 and 62 genes that are cited in the literature, respectively. The first analysis seems to report more known genes but the three analyses obtain comparable results, by finding in the literature more than a half of the genes selected by the Shapley value.

Moreover, a study based on *Reactome* (Croft and et al. 2004) was performed in order to compare the three analyses. Reactome is a repository of biological pathways, namely groups of reactions among nucleic acids, proteins and another kind of molecules that interact as part of biological processes as for example the regulation of gene expression, metabolism, etc. The three lists of 100 genes have been analyzed, yielding the following results: the first analysis identifies 51 genes, the second 45 and the third 47 (see the right-hand side of Fig. 10). These results are coherent with the Literature Mining-based results. Moreover, we observe that the first analysis reports 329 pathways, the second 379 and the third 219. This information could indicate that the quality of the genes found by the second analysis is higher that the other two analyses.

The *Network Cancer of Genes* tool (NCG5.0) was used to further investigate the results of the analysis from a biological point of view. This tool only provides information about known cancer genes and it is therefore too restrictive to be used in a quantitative comparison as before. For example, a gene could be relevant as acting as a “switch” of a known oncogene (cancer gene) or co-regulate an important related process but it would not be reported by NGC, unless it is itself an oncogene. However, this tool provides some useful information from a qualitative point of view, allowing us to evaluate the results of our analysis and to compare them on the basis of the information it provides.

The first 100 genes for each analysis in Table 3 have been studied with NCG, with the objective of understanding their biological relevance from a qualitative perspective. The first analysis finds 5 oncogenes, the second 20 and the third 11. These results support the idea that the second analysis reports genes with a higher quality. However, it is important to emphasize that the second analysis uses a priori information, by considering as input 23 well-known lung cancer genes, precisely obtained using NCG. It must be noted that 15 genes out of these 20 were used as input key-genes. Therefore, we argue that each analysis identifies, respectively, 5, 5 and 11 not previously known oncogenes. The first and the third analysis do not use any a priori knowledge. Nevertheless, 4 out of 5 genes obtained by the first analysis are in the well-known set of 23 lung cancer genes, as well as 2 out of 11 genes obtained by the third analysis.

Moreover, it is interesting to further investigate those genes that are reported only by the proposed relevance index but not by the other (classical) centrality measures. With respect to this, the first analysis presents 19 genes, the second 71 and the third 33 that are selected only by the Shapley value. These sets of genes have also been analyzed using the NCG tool. The first analysis does not show any known cancer gene according to the information supported by the tool. However, the second analysis reports 16 of 71 genes as cancer genes and the third analysis 3 of 33. So, the second analysis presents the best results in this sense, but it must be noted that 15 of the 16 genes are precisely part of the 23 lung cancer genes used as a priori information in the second analysis. Therefore, the second analysis only reports 1 cancer gene which is not previously known and used as input.

The cancer genes reported by the second analysis that belongs to the set of key-genes used as input are *ATXN3L*, *CDH10*, *COL11A1*, *DACH1*, *DNAH3*, *FGFR4*, *GRM8*, *HLA*-*A*, *NRAS*, *PAK*3, *PDIA*4, *PPP*1*R*3*A*, *PTPRD*, *RUNX*1*T*1, and *ZMYND*10. The gene *G*6*PC* is also reported by the second analysis but it is not included in the input set of key genes. This gene is a liver cancer gene with a functionality related to the regulation of intracellular processes and metabolism. Furthermore, Table 5 shows the cancer genes reported by the third analysis. It can be observed that they are leukemia, lung and glioblastoma cancer genes. The gene *CD*1*B* is a lung cancer that belongs to the set of key genes used in the second analysis. It is important to note that this a priori information is not used in the third analysis.

**Table 5:** Third analysis: cancer genes reported only by the Shapley value (and not by other centrality measures)

Gene	Protein function	Properties	Primary	Cancer
name			site	type
GNAT1	Cell response to stimuli/signal transduction	Interaction with 5 proteins and it’s part of a complex	Blood	Leukemia
*CD1B*	This gene has no functional information	Interaction with 2 proteins and it’s part of a complex	Lung	Lung
GML	Cell cycle/regulation of intracellular processes and metabolism/signal transduction	Interaction with proteins	Brain	Glioblastoma

## Conclusions

In this paper, we proposed a relevance index for nodes in gene co-expression networks, with the objective of measuring the potential of genes in acting as intermediaries between hub nodes and leaf nodes and preserving the regulatory activity within gene networks. For this purpose, we used a game-theoretic approach, by defining a cooperative game where the strength of a coalition of genes depends on the a priori importance of the genes in its neighborhood. The Shapley value of such a game is proposed as a new relevance index for genes. Our methodology is supported by a property-driven approach, where the set of properties satisfied by the Shapley value have a biological interpretation. Moreover, an experimental study is conducted on a gene expression dataset from microarray technology, related to a lung cancer disease and the results of the Shapley value are compared with classical centrality measures.

The versatility of the relevance index and its very low computational complexity ($\mathcal {O}(|N|+|E|$) allow the combination of a game-theoretical approach with other techniques from network analysis. Indeed, we used an algorithm from cluster analysis that identifies overlapping clusters of genes, in order to assess the a priori importance of genes in the network under analysis. An interesting direction for future research is the further study of these techniques, in order to refine the relevance analysis, and the application of our model to other gene networks in order to provide new biological knowledge.

We want to emphasize here that we cannot expect that a single relevance index, characterized by a so low complexity, could capture all possible critical aspects of the problem. As it often happens in the property-driven analysis of centrality measures, natural properties satisfied by an index in a given class of graphs may be outweighed by a less intuitive property of the same index in a different family of graphs. This is the case, for instance, of dense graphs or graphs with a relevant number of cliques, as the one considered in Example 2. Even if the interpretation of the ranking of nodes of Example 2 is coherent with an evaluation of the leader nodes in a community structure (Li and Daniels 2015; Li et al. 2015, 2018), the interpretation of the Shapley value along the lines discussed in the motivating example of “A motivating example” section and in Example 1, does not apply to the network of Fig. 2. Even if many biological networks, such as the co-expression networks examined in this work (recall that, as described in “Description of the dataset” section, a network is characterized by an equilibrium between connectivity and sparsification (Christiano Silva and Zhao 2016)), contain more substructure resembling the graph of Fig. 1 than resembling the one of Fig. 2, a precise characterization of the class of graphs where the interpretation of the Shapley value provided in “A motivating example” section applies, is still an open question and an interesting issue for future research.

## Additional files

Additional file 1 Table S1: Genes selected by *ρ* (first analysis) (PDF 65 kb)

Additional file 2 Table S2: Genes selected by *ρ* (second analysis) (PDF 54 kb)

Additional file 3 Table S3: Genes selected by *ρ* (third analysis) (PDF 64 kb)

## Abbreviations

GEO: Gene expression omnibus
NCBI: National center for biotechnology information
NCG: Network cancer of genes
