# Supplementary material for: An application of the Shapley value to the analysis of co-expression networks
Source: Appl Netw Sci. 2018 Aug 24;3(1):35. doi: 10.1007/s41109-018-0095-y (PMC6214322; doi:10.1007/s41109-018-0095-y)
Supplement: Supplementary file 1 — Table S1: Genes selected by ρ (first analysis) (PDF 65 kb) [file 41109_2018_95_MOESM1_ESM.pdf]

S1 Table

Gene symbol	Gene name	ρ
ZNF771[1,2,3]	zinc finger protein 771	11.9992275312791
ATP2B2[1,2,3]	ATPase plasma membrane Ca2+ transporting 2	11.2142270966438
CDHR5[1,2,3]	cadherin related family member 5	9.1885254409648
WNT10B[2,3]	Wnt family member 10B	8.13030213112638
PRB1[1,2,3]	proline rich protein BstNI subfamily 1	8.00968140817491
SEZ6L[2,3]	seizure related 6 homolog like	7.91259620038736
C1orf105[1,3]	chromosome 1 open reading frame 105	7.3200847410164
HOXC8[1,2,3]	homeobox C8	7.23088256293767
TSPY1[1,2,3]	testis specific protein, Y-linked 1	6.83961493570101
EMX1[2,3]	empty spiracles homeobox 1	6.82705292482686
EDA[1,2,3]	ectodysplasin A	6.79840320069657
TCL6[1,2,3]	T-cell leukemia/lymphoma 6 (non-protein coding)	6.20557451207543
CCL25[1,2,3]	C-C motif chemokine ligand 25	6.16485389207215
MRPL3[3]	mitochondrial ribosomal protein L3	6.14618678301022
ZNF335[3]	zinc finger protein 335	5.99469477265226
GRIN1[2,3]	glutamate ionotropic receptor NMDA type subunit 1	5.86845584630501
PAX8[1,2,3]	paired box 8	5.70669682587376
FOXL1[2,3]	forkhead box L1	5.58403737718699
PDIA2[3]	protein disulfide isomerase family A member 2	5.53122000122485
AK024615[1,3]	NA	5.50876181959234
GYPA[1,2,3]	glycophorin A (MNS blood group)	5.22594690710353
NME1[3]	NME/NM23 nucleoside diphosphate kinase 1	5.08064008570667
CDK1[1,3]	cyclin dependent kinase 1	4.91327186640558
D16471[3]	NA	4.83935622133443
CCT3[3]	chaperonin containing TCP1 subunit 3	4.6954499389479
KCNB2[1,2,3]	potassium voltage-gated channel subfamily B member 2	4.69272227270457
PRB4[1,2,3]	proline rich protein BstNI subfamily 4	4.60403627742111
TCF21[1,3,4]	transcription factor 21	4.60089382572832
BMP8B[1,2,3]	bone morphogenetic protein 8b	4.59970896117806
FHL1[1,4]	four and a half LIM domains 1	4.52930361513385
LDB2[1,3,4]	LIM domain binding 2	4.51187237653154
EDNRB[1,4]	endothelin receptor type B	4.47714205047538
FAM107A[1,3,4]	family with sequence similarity 107 member A	4.34816411415173
TEK[1,3,4]	TEK receptor tyrosine kinase	4.11666304132315
MAP3K19[2,3]	mitogen-activated protein kinase kinase kinase 19	3.97408038386278
CDC20	cell division cycle 20	3.93354962423571
LLGL1[2,3]	LLGL1, scribble cell polarity complex component	3.89338477336018
JAM2[1,4]	junctional adhesion molecule 2	3.83394418115792
GRK5[4]	G protein-coupled receptor kinase 5	3.79630010066131
AK025422[1,3]	NA	3.7846980734651
CA4[1,4]	carbonic anhydrase 4	3.77498362077175

HAPLN2[2,3]	hyaluronan and proteoglycan link protein 2	3.74556662369419
FLJ11292[2,3]	NA	3.7060244652241
AK022038[2,3]	NA	3.67100666739232
HMX1[3]	H6 family homeobox 1	3.64186374038613
ARVCF[3]	armadillo repeat gene deleted in velocardiofacial syndrome	3.5586929254162
CKS1B	CDC28 protein kinase regulatory subunit 1B	3.54580594585801
CD93[1,4]	CD93 molecule	3.51587786313662
FIGF[1,4]	vascular endothelial growth factor D	3.50044064981034
ADAM5	ADAM metallopeptidase domain 5 (pseudogene)	3.49240648489671
KPNA2[3]	karyopherin subunit alpha 2	3.48841969287436
TOP2A[3]	topoisomerase (DNA) II alpha	3.47668462623347
AK025072[3]	NA	3.46433746454934
SASH1[1,4]	SAM and SH3 domain containing 1	3.44723545921552
KIR2DS3[2,3]	killer cell immunoglobulin like receptor, two Ig domains and short cytoplasmic tail 3	3.44338855236704
AOC3[1,3,4]	amine oxidase, copper containing 3	3.4212214776578
FXYD6[1,4]	FXYD domain containing ion transport regulator 6	3.41401394751061
BTNL8	butyrophilin like 8	3.41298981322471
RAMP2[1,4]	receptor activity modifying protein 2	3.40102612306597
AGER[1,4]	advanced glycosylation end product-specific receptor	3.38854823247169
HTR3B[2,3]	5-hydroxytryptamine receptor 3B	3.38731134451332
KIR2DS1[2,3]	killer cell immunoglobulin like receptor, two Ig domains and short cytoplasmic tail 1	3.38492496733694
CD53	CD53 molecule	3.37511461457082
CLEC3B[1,3,4]	C-type lectin domain family 3 member B	3.36879474738695
DLGAP5	DLG associated protein 5	3.36645806294723
STARD13[1,4]	StAR related lipid transfer domain containing 13	3.35970120523624
OR1A1	olfactory receptor family 1 subfamily A member 1	3.35835999448623
RAX [2,3]	retina and anterior neural fold homeobox	3.34890361540423
LIMS2[1,4]	LIM zinc finger domain containing 2	3.33945435405761
MIF	macrophage migration inhibitory factor (glycosylation-inhibiting factor)	3.30746031746032
CHRM2	cholinergic receptor muscarinic 2	3.28992041717679
AW975117	NA	3.27085349169641
PECAM1[1,4]	platelet and endothelial cell adhesion molecule 1	3.27036184818285
NODAL[3]	nodal growth differentiation factor	3.26050389651186
FOXF1[1,4]	forkhead box F1	3.26006295780186
HRG[1,2,3]	histidine rich glycoprotein	3.25133676965899
CAMK1G[2,3]	calcium/calmodulin dependent protein kinase IG	3.24179862903933
KCNJ1	potassium voltage-gated channel subfamily J member 1	3.23365890625387
VSIG4[3]	V-set and immunoglobulin domain containing 4	3.18251984492977
SMCP[2,3]	sperm mitochondria associated cysteine rich protein	3.17712705414093
AF119878[2]	NA	3.17415257308162
ILF2	interleukin enhancer binding factor 2	3.16155261732781

MAD2L1	MAD2 mitotic arrest deficient-like 1 (yeast)	3.15320581892578
DLX2[2,3]	distal-less homeobox 2	3.07425506253775
FUT7[3]	fucosyltransferase 7	3.07120067035173
MARCO[3]	macrophage receptor with collagenous structure	3.05487810062594
RAI2[1,4]	retinoic acid induced 2	3.05452583812376
AK022363[2,3]	NA	3.05450798012059
MFAP4[1,3]	microfibrillar associated protein 4	3.04897947000773
GHRHR[3]	growth hormone releasing hormone receptor	3.00490573526143
ACADL[1,4]	acyl-CoA dehydrogenase, long chain	2.99154511559473
AL162040	NA	2.99086383959853
SLC22A11[2,3]	solute carrier family 22 member 11	2.979451941982
DUX4L2	double homeobox 4 like 2	2.97916410135979
SERPINB10[2]	serpin family B member 10	2.96320258039237
GRM1 [2,3]	glutamate metabotropic receptor 1	2.9593734805239
ABCA8[1,4]	ATP binding cassette subfamily A member 8	2.95599380651336
S1PR1[1,4]	sphingosine-1-phosphate receptor 1	2.92469788005853
SNRPG[3]	small nuclear ribonucleoprotein polypeptide G	2.9132885460193
KCNA2	potassium voltage-gated channel subfamily A member 2	2.9121553250216
TGFBR3[1,4]	transforming growth factor beta receptor 3	2.90053579900917
OPRM1[2,3]	opioid receptor mu 1	2.89181056116898
ATP2B3[2,3]	ATPase plasma membrane Ca2+ transporting 3	2.87401927974233
PART1	prostate androgen-regulated transcript 1 (non-protein coding)	2.85975828610126
PAICS	phosphoribosylaminoimidazole carboxylase; phosphoribosylaminoimidazolesuccinocarboxamide synthase	2.82590246660617
SNRPE	small nuclear ribonucleoprotein polypeptide E	2.82412666489096
RFC4	replication factor C subunit 4	2.79219066632501
GPM6A[1,4]	glycoprotein M6A	2.78089283215017
