# Supplementary material for: An application of the Shapley value to the analysis of co-expression networks
Source: Appl Netw Sci. 2018 Aug 24;3(1):35. doi: 10.1007/s41109-018-0095-y (PMC6214322; doi:10.1007/s41109-018-0095-y)
Supplement: Supplementary file 2 — Table S2: Genes selected by ρ (second analysis) (PDF 54 kb) [file 41109_2018_95_MOESM2_ESM.pdf]

S2 Table

Input	Approved name	Shapley.value.2.	k
MRPL42	mitochondrial ribosomal protein L42	0.5	0
NRAS	neuroblastoma RAS viral oncogene homolog	0.5	1
PDIA4	protein disulfide isomerase family A member 4	0.5	1
PRDX4	peroxiredoxin 4	0.5	0
DACH1	dachshund family transcription factor 1	0.404807256235828	1
RUNX1T1	RUNX1 translocation partner 1	0.388888888888889	1
SEMA6A	semaphorin 6A	0.388888888888889	0
ATP2B2[1,2,3]	ATPase plasma membrane Ca2+ transporting 2	0.379710975299211	0
AK024553	NA	0.361204481792717	0
CDH10	cadherin 10	0.333333333333333	1
GC	GC, vitamin D binding protein	0.333333333333333	0
HLA-A	major histocompatibility complex, class I, A	0.333333333333333	1
HLA-C	major histocompatibility complex, class I, C	0.333333333333333	0
HLA-F	major histocompatibility complex, class I, F	0.333333333333333	0
SCGN	secretagogin, EF-hand calcium binding protein	0.333333333333333	0
DLX2[2,3]	distal-less homeobox 2	0.290909090909091	0
PRB1[1,2,3]	proline rich protein BstNI subfamily 1	0.270887445887446	0
GYPA[1,2,3]	glycophorin A (MNS blood group)	0.263044308632544	0
KCNJ1	potassium voltage-gated channel subfamily J member 1	0.236853832442068	0
STARD13[1,4]	StAR related lipid transfer domain containing 13	0.214331065759637	0
CA1[3]	carbonic anhydrase 1	0.20952380952381	0
CDHR5[1,2,3]	cadherin related family member 5	0.2	0
GPATCH4	G-patch domain containing 4	0.2	0
GRM8	glutamate metabotropic receptor 8	0.2	1
SEZ6L[1,2,3]	seizure related 6 homolog like	0.2	0
DSCAM[1,2]	DS cell adhesion molecule	0.1994588744458874	0
KCNS1[2]	potassium voltage-gated channel modifier subfamily S member 1	0.178030303030303	0
OR1A1	olfactory receptor family 1 subfamily A member 1	0.170887445887446	0
SLC12A5	solute carrier family 12 member 5	0.170887445887446	0
C9orf116	chromosome 9 open reading frame 116	0.166666666666667	0
CFAP45	cilia and flagella associated protein 45	0.166666666666667	0
DNAI1	dynein axonemal intermediate chain 1	0.166666666666667	0
TEKT2	tektin 2	0.166666666666667	0
ZBBX	zinc finger B-box domain containing	0.166666666666667	0
ZMYND10	zinc finger MYND-type containing 10	0.166666666666667	1
AW975117	NA	0.15952380952381	0
HRG[1,2,3]	histidine rich glycoprotein	0.158282403870639	0

AL162040	NA	0.149732620320856	0
AK025352[2]	NA	0.149458874458874	0
BTNL8	butyrophilin like 8	0.149458874458874	0
CAMK1G[2,3]	calcium/calmodulin dependent protein kinase IG	0.149458874458874	0
CDKL2	cyclin dependent kinase like 2	0.149458874458874	0
IFNA13[2]	interferon alpha 13	0.149458874458874	0
FEZ1	fasciculation and elongation protein zeta 1	0.148571428571429	0
PARVA	parvin alpha	0.148571428571429	0
ATXN3L	ataxin 3 like	0.142857142857143	1
CLEC1A	C-type lectin domain family 1 member A	0.142857142857143	0
DNAH3	dynein axonemal heavy chain 3	0.142857142857143	1
NXPE4	neurexophilin and PC-esterase domain family member 4	0.142857142857143	0
PKNOX2	PBX/knotted 1 homeobox 2	0.142857142857143	0
PRO2958	NA	0.142857142857143	0
PTPRD	protein tyrosine phosphatase, receptor type D	0.142857142857143	0
ROR1	receptor tyrosine kinase like orphan receptor 1	0.142857142857143	0
SCAND2P[2]	SCAN domain containing 2 pseudogene	0.142857142857143	0
CCL25[1,2,3]	C-C motif chemokine ligand 25	0.128030303030303	0
TTTY1B	testis-specific transcript, Y-linked 1B (non-protein coding)	0.121428571428571	0
BC000772[2]	NA	0.120187165775401	0
DLGAP1	DLG associated protein 1	0.111363636363636	0
PYY2[2]	peptide YY, 2 (pseudogene)	0.111363636363636	0
KRT12	keratin 12	0.107575757575758	0
C16orf72	chromosome 16 open reading frame 72	0.1	0
PAK3	p21 (RAC1) activated kinase 3	0.1	1
ZNF442	zinc finger protein 442	0.0994588744588745	0
AK025038	NA	0.0909090909090909	0
AK025430	NA	0.0909090909090909	0
C9orf38	NA	0.0909090909090909	0
COL10A1	collagen type X alpha 1 chain	0.0909090909090909	0
COL11A1	collagen type XI alpha 1 chain	0.0909090909090909	1
COL1A1	collagen type I alpha 1	0.0909090909090909	0
COL5A2	collagen type V alpha 2 chain	0.0909090909090909	0
D16471[3]	NA	0.0909090909090909	0
FAP	fibroblast activation protein alpha	0.0909090909090909	0
G6PC	glucose-6-phosphatase catalytic subunit	0.0909090909090909	0
GREM1	gremlin 1, DAN family BMP antagonist	0.0909090909090909	0
LRRC15	leucine rich repeat containing 15	0.0909090909090909	0
MTMR7	myotubularin related protein 7	0.0909090909090909	0
MXRA5	matrix remodeling associated 5	0.0909090909090909	0
PPP1R3A	protein phosphatase 1 regulatory subunit 3A	0.0909090909090909	1
SPP1[3]	secreted phosphoprotein 1	0.0909090909090909	0
SULF1[3]	sulfatase 1	0.0909090909090909	0

TEX41	testis expressed 41 (non-protein coding)	0.0909090909090909	0
THBS2	thrombospondin 2	0.0909090909090909	0
BMP8B[1,2,3]	bone morphogenetic protein 8b	0.0868538324420677	0
C1orf105[1,3]	chromosome 1 open reading frame 105	0.0868538324420677	0
CSRP3	cysteine and glycine rich protein 3	0.0868538324420677	0
CPN2	carboxypeptidase N subunit 2	0.0827922077922078	0
GLRA2	glycine receptor alpha 2	0.0827922077922078	0
AAK1[2,3]	AP2 associated kinase 1	0.078030303030303	0
KCNA2	potassium voltage-gated channel subfamily A member 2	0.078030303030303	0
TCL6[1,2,3]	T-cell leukemia/lymphoma 6 (non-protein coding)	0.078030303030303	0
LOC51145	NA	0.0754901960784314	0
CLDN18	claudin 18	0.0714739229024943	0
ERG[1,4]	ERG, ETS transcription factor	0.0714739229024943	0
FGFR4[4]	fibroblast growth factor receptor 4	0.0714739229024943	0
FOXF1[1,4]	forkhead box F1	0.0714739229024943	0
GPM6A[1,4]	glycoprotein M6A	0.0714739229024943	0
GRK5[1]	G protein-coupled receptor kinase 5	0.0714739229024943	0
KCNK3[1]	potassium two pore domain channel subfamily K member 3	0.0714739229024943	0
LRRN3[1,4]	leucine rich repeat neuronal 3	0.0714739229024943	0
SASH1[1,4]	SAM and SH3 domain containing 1	0.0714739229024943	0
SLIT2[1,4]	slit guidance ligand 2	0.0714739229024943	0
TEK[1,3,4]	TEK receptor tyrosine kinase	0.0714739229024943	0
TGFBR3[1,4]	transforming growth factor beta receptor 3	0.0714739229024943	0
CASR	calcium sensing receptor	0.0714285714285714	0
CST8	cystatin 8	0.0714285714285714	0
CTNNA3	catenin alpha 3	0.0714285714285714	0
CCKBR	cholecystokinin B receptor	0.0701871657754011	0
GRM1[2,3]	glutamate metabotropic receptor 1	0.0701871657754011	0
