# Supplementary material for: An application of the Shapley value to the analysis of co-expression networks
Source: Appl Netw Sci. 2018 Aug 24;3(1):35. doi: 10.1007/s41109-018-0095-y (PMC6214322; doi:10.1007/s41109-018-0095-y)
Supplement: Supplementary file 3 — Table S3: Genes selected by ρ (third analysis) (PDF 64 kb) [file 41109_2018_95_MOESM3_ESM.pdf]

S3 Table

Gene symbol	Gene name	ρ	k
ATP2B2[1,2,3]	ATPase plasma membrane Ca2+ transporting 2	18.4968930265747	8
C1orf105[1,3]	chromosome 1 open reading frame 105	13.7288298097441	8
PRB1[1,2,3]	proline rich protein BstNI subfamily 1	13.3347102121442	3
CCL25[1,2,3]	C-C motif chemokine ligand 25	12.9864672408187	10
CDHR5[1,3]	cadherin related family member 5	12.4145709883877	3
PDIA2[3]	protein disulfide isomerase family A member 2	11.6061057741986	5
GYPA[1,2,3]	glycophorin A (MNS blood group)	11.5052460543739	8
HOXC8[1,2,3]	homeobox C8	10.8814446249438	4
TCL6[1,2,3]	T-cell leukemia/lymphoma 6 (non-protein coding)	10.8729995724874	3
TSPY1[1,2,3]	testis specific protein, Y-linked 1	10.4394568980079	3
HRG[1,2,3]	histidine rich glycoprotein	10.0102747292022	10
MLXIPL[3]	MLX interacting protein like	9.83327153093983	9
EDA[1,2,3]	ectodysplasin A	9.64847640412969	3
SEZ6L[1,2,3]	seizure related 6 homolog like	9.47867509832141	3
OR1A1	olfactory receptor family 1 subfamily A member 1	9.33046614911308	7
AK024615[1,2,3]	NA	9.22821633760145	8
PRB4[1,2,3]	proline rich protein BstNI subfamily 4	9.11861977475885	3
ZNF335[3]	zinc finger protein 335	9.09897657855088	4
KCNA2	potassium voltage-gated channel subfamily A member 2	8.86054243002737	7
ZNF771[1,2,3]	zinc finger protein 771	8.81703058371348	3
AK025072[3]	NA	8.72320592268589	7
LOC100506002	NA	8.61427535284604	11
AF119878	NA	8.56221670492298	7
BMP8B[1,2,3]	bone morphogenetic protein 8b	8.51405007440748	8
NODAL[3]	nodal growth differentiation factor	8.4558549181182	7
ELAVL3[3]	ELAV like neuron-specific RNA binding protein 3	8.44550175908849	7
EMX1[1,2,3]	empty spiracles homeobox 1	8.4242643003209	3
DSCAM[2]	DS cell adhesion molecule	8.16553943246246	10
HMX1[3]	H6 family homeobox 1	8.15525277481443	5
KCNJ1	potassium voltage-gated channel subfamily J member 1	8.13025327936225	8
CAMK1G[2,3]	calcium/calmodulin dependent protein kinase IG	7.78314620880678	6
LTB4R2	leukotriene B4 receptor 2	7.71255655866246	10
BTNL8	butyrophilin like 8	7.64311215670017	8
HAPLN2[2,3]	hyaluronan and proteoglycan link protein 2	7.63529888053862	3
FUT7[3]	fucosyltransferase 7	7.50677480941965	7

ADAM5	ADAM metallopeptidase domain 5 (pseudogene)	7.42666528314341	8
IFNA13[2]	interferon alpha 13	7.40319722519809	7
CDKL2	cyclin dependent kinase like 2	7.31993815928898	7
MAP3K19[2,3]	mitogen-activated protein kinase kinase kinase 19	7.23211910217063	4
PAX8[1,2,3]	paired box 8	7.11851211195136	3
CA14	carbonic anhydrase 14	7.10052407649743	7
MYL1	myosin light chain 1	7.09947777830419	8
EPO	erythropoietin	7.09189491213324	6
KCNB2[1,2,3]	potassium voltage-gated channel subfamily B member 2	7.07536136721792	3
FOXL1[2,3]	forkhead box L1	6.95024136477208	4
AAK1[2,3]	AP2 associated kinase 1	6.91822837078907	7
BC000772[2]	NA	6.88184604860484	7
GNAT1	G protein subunit alpha transducin 1	6.81948519912769	10
ZNF442	zinc finger protein 442	6.81754467719459	8
FEV[2]	FEV, ETS transcription factor	6.79739185238554	7
LDB2[1,3,4]	LIM domain binding 2	6.78913784624733	1
DUX4L2	double homeobox 4 like 2	6.74419555996507	6
ARVCF[3]	armadillo repeat gene deleted in velocardiofacial syndrome	6.73928464890796	8
GRM1[2,3]	glutamate metabotropic receptor 1	6.68057522752628	5
LOC51145	NA	6.65805138335087	7
FLJ11292[2,3]	NA	6.57372359719334	4
WT1-AS[2]	WT1 antisense RNA	6.53248118174916	7
TEK[1,3,4]	TEK receptor tyrosine kinase	6.46678086791464	1
CA1[3]	carbonic anhydrase 1	6.44871559296281	7
CCDC70[2]	coiled-coil domain containing 70	6.41209424073279	7
GPR135[2,3]	G protein-coupled receptor 135	6.35701222209935	6
GP1BB	glycoprotein Ib platelet beta subunit	6.29699941893655	6
GPR144	adhesion G protein-coupled receptor D2	6.28198739685931	6
AW975117	NA	6.15144694479165	7
SCN10A[2]	sodium voltage-gated channel alpha subunit 10	6.12969729143662	7
GPR52	G protein-coupled receptor 52	6.12025864336093	7
DOHH	deoxyhypusine hydroxylase/monooxygenase	6.07302532724421	6
ATP2B3[2,3]	ATPase plasma membrane Ca2+ transporting 3	6.04233529409678	4
WNT10B[2,3]	Wnt family member 10B	5.98285094528619	3
CD1B	CD1b molecule	5.93382563986049	7
TCF21[1,3,4]	transcription factor 21	5.91568960918555	1
SLC17A4	solute carrier family 17 member 4	5.89244118922335	8
NR6A1[1]	nuclear receptor subfamily 6 group A member 1	5.88146534927107	5
AF023203	NA	5.86486783569472	5
GML	glycosylphosphatidylinositol anchored molecule like	5.85409440585522	7

KCNS1[2]	potassium voltage-gated channel modifier subfamily S member 1	5.84452694533979	8
LLGL1[2,3]	LLGL1, scribble cell polarity complex component	5.8323488700226	3
CENPO	centromere protein O	5.82700828006012	4
EDNRB[1]	endothelin receptor type B	5.79607391080148	1
CSRP3	cysteine and glycine rich protein 3	5.7044271478122	7
CLEC3B[1,3,4]	C-type lectin domain family 3 member B	5.64384144092779	1
MARCO[3]	macrophage receptor with collagenous structure	5.61624750262895	4
AK025352[2]	NA	5.61050911390812	4
JAM2[1,4]	junctional adhesion molecule 2	5.5894640710116	1
BMP15	bone morphogenetic protein 15	5.58487772585608	7
NPR1[3]	natriuretic peptide receptor 1	5.58067578760658	5
AK024553	NA	5.56052450889608	7
FHL1[1,4]	four and a half LIM domains 1	5.53317512915856	1
SASH1[1,4]	SAM and SH3 domain containing 1	5.51964156610769	2
AOC3[1,3,4]	amine oxidase, copper containing 3	5.51219671063685	1
CYP11B2[3]	cytochrome P450 family 11 subfamily B member 2	5.49779257564747	6
TNFRSF13B	TNF receptor superfamily member 13B	5.48285549331722	9
AK025422[1,2,3]	NA	5.46235640137296	4
SLC14A2[2]	solute carrier family 14 member 2	5.45679545178622	3
RUNX2[2]	runt related transcription factor 2	5.43705868607741	3
DLX2[2,3]	distal-less homeobox 2	5.42927250433307	3
FNDC8[2,3]	fibronectin type III domain containing 8	5.42575479256574	5
FIGF[1,4]	vascular endothelial growth factor D	5.38818797984467	1
PART1	prostate androgen-regulated transcript 1 (non-protein coding)	5.35987464820128	6
SLC12A5	solute carrier family 12 member 5	5.28505220194836	10
CCRN4L[2,3]	nocturnin	5.26296832657673	4
HTR3B[2,3]	5-hydroxytryptamine receptor 3B	5.23677967416225	4
VSIG4[3]	V-set and immunoglobulin domain containing 4	5.22895709127482	3
ASB4	ankyrin repeat and SOCS box containing 4	5.22440764676332	4
SLC22A11[2,3]	solute carrier family 22 member 11	5.19817921281046	3
SERPINB10[2]	serpin family B member 10	5.15717776478373	2
OR1D2[2]	olfactory receptor family 1 subfamily D member 2	5.14529200069891	4
OR10C1	olfactory receptor family 10 subfamily C member 1 (gene/pseudogene)	5.13923156921155	5
